# Supplementary figures and images for: Predicting the infecting dengue serotype from antibody titre data using machine learning
Source: PLoS Comput Biol. 2024 Dec 23;20(12):e1012188. doi: 10.1371/journal.pcbi.1012188 (PMC11706371; doi:10.1371/journal.pcbi.1012188)

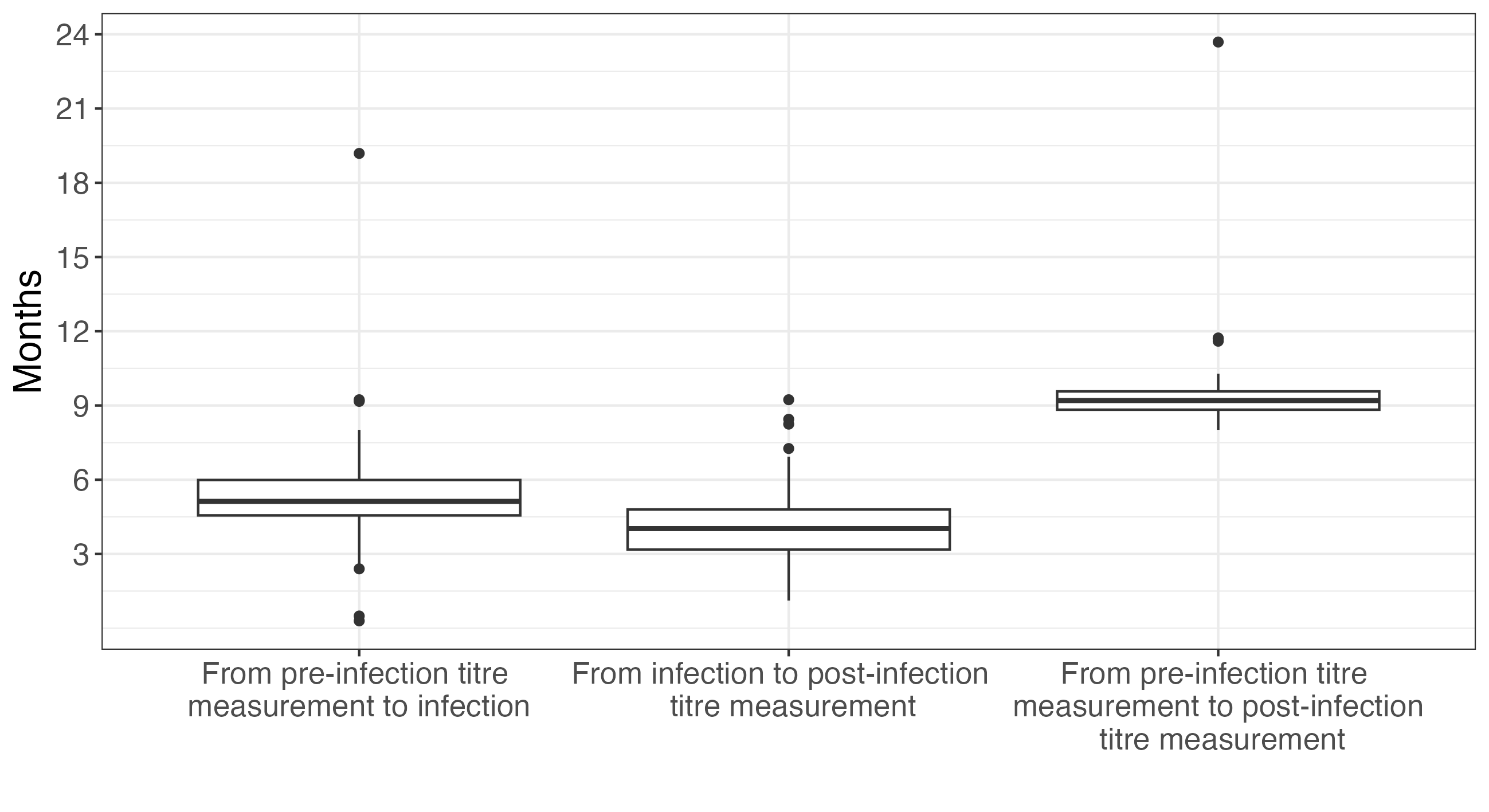

Supplement: S2 Fig — Boxes represent the 25th to the 75th percentile of each group’s distribution of values, the horizonal line represents the median, and the upper and lower whiskers denote the most extreme values within 1.5 interquartile range of the 25th and 75th percentile of each group. Data beyond the whiskers are dented outliers and are plotted individually as points. PRNT: plaque reduction neutralisation test. (TIF) [file pcbi.1012188.s002.tif]

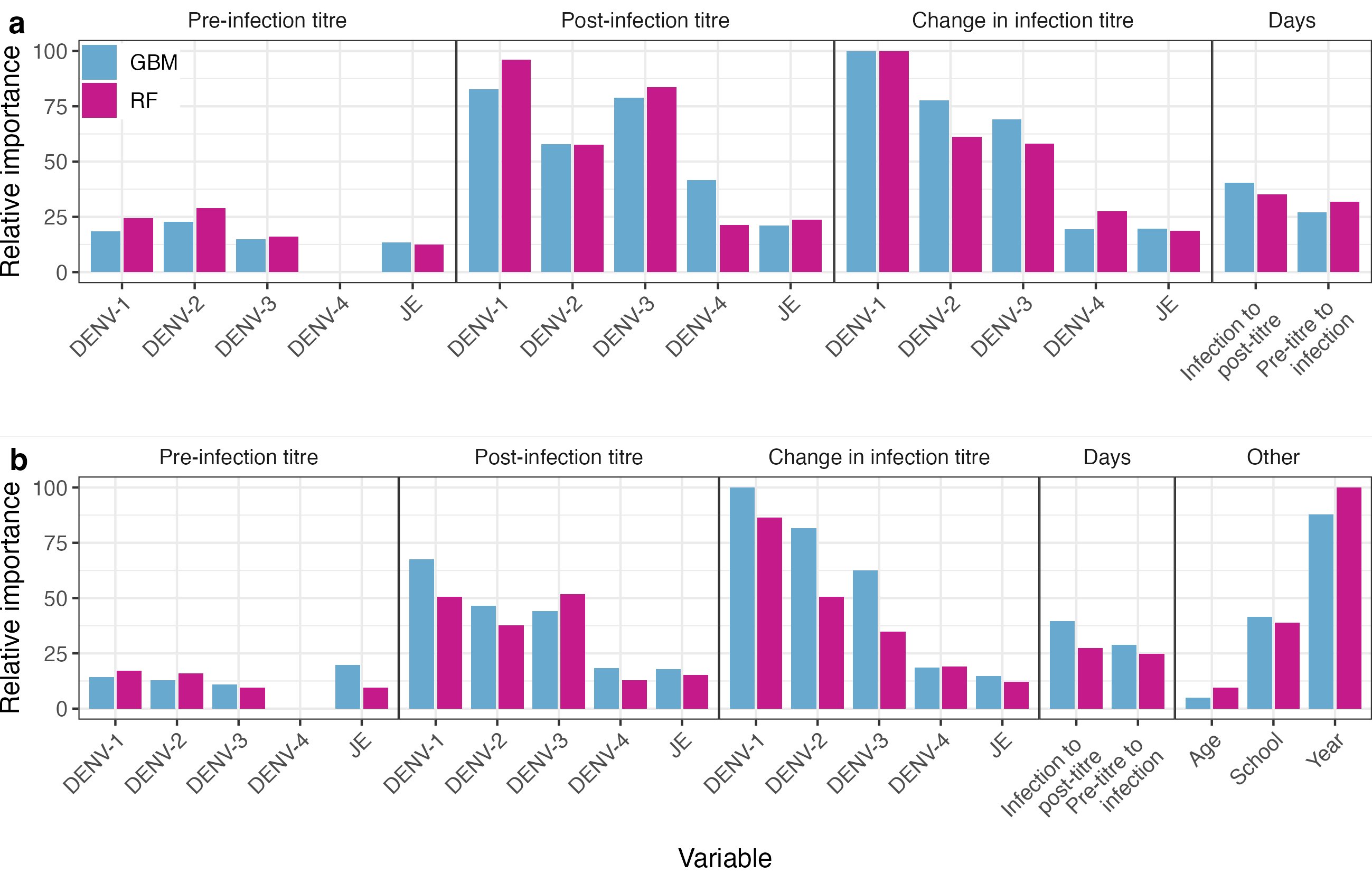

Supplement: S4 Fig — Variable importance of each model in predicting the infecting serotype was calculated using leave-one-out cross validation on the full dataset. Scenario A: all titre predictor variables (pre- and post-infection PRNT titres and change in titre against all four-dengue virus (DENV) serotypes and Japanese encephalitis virus (JEV), and the number of days between measurement of the pre- and post-infection titres and the date of infection). Scenario B: titre predictor variables plus age, year of infection, and school. GBM: gradient boosting machine. RF: random forest. PRNT: plaque reduction neutralisation test. (TIF) [file pcbi.1012188.s004.tif]
