# Supplementary material for: Predicting the infecting dengue serotype from antibody titre data using machine learning
Source: PLoS Comput Biol. 2024 Dec 23;20(12):e1012188. doi: 10.1371/journal.pcbi.1012188 (PMC11706371; doi:10.1371/journal.pcbi.1012188)
Supplement: S1 Table — Note, DENV seronegative children are by definition those with a pre-infection titre <10 for all four serotypes. DENV: dengue virus. JEV: Japanese encephalitis virus. (DOCX) [file pcbi.1012188.s006.docx]

**S1 Table: Individual pre- (JEV) and post-infection neutralising antibody titres in DENV seronegative children whose highest post-infection titre did not match the infecting serotype.** Note, DENV seronegative children are by definition those with a pre-infection titre <10 for all four serotypes. DENV: dengue virus. JEV: Japanese encephalitis virus.

| Infecting serotype (RT-PCR) | Pre-infection JEV | Post-infection DENV-1 | Post-infection DENV-2 | Post-infection DENV-3 | Post-infection DENV-4 | Post-infection JEV |
| --- | --- | --- | --- | --- | --- | --- |
| DENV-1 | 1.28 | 3.20 | 3.20 | 3.26 | 2.01 | 1.88 |
|  | 0.00 | 2.60 | 2.70 | 2.77 | 0.00 | 1.51 |
|  | 1.51 | 2.86 | 2.68 | 2.86 | 0.00 | 1.79 |
|  | 0.00 | 2.97 | 2.72 | 3.25 | 0.00 | 1.36 |
| DENV-2 | 2.86 | 2.17 | 2.87 | 2.97 | 0.00 | 2.85 |
|  | 1.68 | 1.61 | 2.91 | 3.06 | 0.00 | 1.89 |
|  | 1.11 | 2.46 | 2.98 | 3.01 | 1.36 | 1.62 |
|  | 1.23 | 2.18 | 2.41 | 2.44 | 0.00 | 1.30 |
|  | 0.00 | 2.37 | 2.81 | 3.05 | 1.67 | 0.00 |
| DENV-3 | 1.72 | 3.89 | 3.54 | 3.74 | 1.34 | 2.01 |
|  | 1.49 | 4.01 | 2.71 | 3.88 | 0.00 | 1.77 |
