# Supplementary material for: Predicting the infecting dengue serotype from antibody titre data using machine learning
Source: PLoS Comput Biol. 2024 Dec 23;20(12):e1012188. doi: 10.1371/journal.pcbi.1012188 (PMC11706371; doi:10.1371/journal.pcbi.1012188)
Supplement: S2 Table — Note, DENV seronegative children are by definition those with a pre-infection titre <10 for all four serotypes. DENV: dengue virus. JEV: Japanese encephalitis virus. (DOCX) [file pcbi.1012188.s007.docx]

**S2 Table: Individual pre- (JEV) and post-infection neutralising antibody titres in DENV seronegative children whose highest post-infection titre matched the infecting serotype.** Note, DENV seronegative children are by definition those with a pre-infection titre <10 for all four serotypes**.** DENV: dengue virus. JEV: Japanese encephalitis virus.

| Infecting serotype (RT-PCR) | Pre-infection JEV | Post-infection DENV-1 | Post-infection DENV-2 | Post-infection DENV-3 | Post-infection DENV-4 | Post-infection JEV |
| --- | --- | --- | --- | --- | --- | --- |
| DENV-1 | 0.00 | 2.74 | 2.58 | 2.63 | 1.98 | 0.00 |
|  | 1.85 | 2.69 | 2.54 | 2.68 | 0.00 | 1.96 |
|  | 0.00 | 2.92 | 1.64 | 2.09 | 0.00 | 0.00 |
|  | 0.00 | 2.22 | 0.00 | 0.00 | 0.00 | 0.00 |
|  | 0.00 | 2.88 | 1.56 | 2.17 | 0.00 | 0.00 |
| DENV-2 | 1.15 | 1.28 | 2.62 | 1.32 | 0.00 | 1.43 |
|  | 1.83 | 1.98 | 2.99 | 2.44 | 0.00 | 2.05 |
|  | 0.00 | 2.91 | 3.04 | 2.59 | 0.00 | 1.15 |
|  | 0.00 | 0.00 | 2.51 | 1.74 | 0.00 | 0.00 |
|  | 2.72 | 2.04 | 2.74 | 1.76 | 1.18 | 2.73 |
|  | 1.18 | 2.09 | 2.97 | 2.75 | 0.00 | 1.68 |
|  | 2.32 | 1.92 | 2.45 | 1.52 | 0.00 | 2.15 |
|  | 1.67 | 2.33 | 2.69 | 2.10 | 0.00 | 2.30 |
| DENV-3 | 0.00 | 3.30 | 3.37 | 3.48 | 1.34 | 1.08 |
|  | 0.00 | 1.76 | 1.52 | 2.75 | 1.62 | 0.00 |
|  | 0.00 | 2.33 | 3.04 | 3.05 | 1.62 | 0.00 |
|  | 2.00 | 0.00 | 1.20 | 3.41 | 0.00 | 2.18 |
|  | 1.56 | 2.05 | 2.64 | 3.00 | 0.00 | 1.81 |
|  | 1.18 | 1.94 | 1.72 | 2.29 | 0.00 | 1.62 |
|  | 1.41 | 2.72 | 2.41 | 3.78 | 0.00 | 1.63 |
|  | 1.76 | 1.97 | 1.43 | 2.12 | 0.00 | 1.63 |
|  | 1.36 | 3.01 | 2.73 | 3.41 | 1.26 | 2.04 |
|  | 1.69 | 2.52 | 2.51 | 3.29 | 0.00 | 2.19 |
