# Supplementary material for: Predicting the infecting dengue serotype from antibody titre data using machine learning
Source: PLoS Comput Biol. 2024 Dec 23;20(12):e1012188. doi: 10.1371/journal.pcbi.1012188 (PMC11706371; doi:10.1371/journal.pcbi.1012188)
Supplement: S3 Table — Seropositive individuals are classified as those with pre-infection titre ≥ 10 for at least one DENV serotype. DENV: dengue virus. JEV: Japanese encephalitis virus. PRNT: plaque reduction neutralisation test. (DOCX) [file pcbi.1012188.s008.docx]

**S3 Table: Modelling scenarios investigated.** Seropositive individuals are classified as those with pre-infection titre $\geq$ 10 for at least one DENV serotype. DENV: dengue virus. JEV: Japanese encephalitis virus. PRNT: plaque reduction neutralisation test.

|  | Scenario A (N=204) | Scenario B (N=204) | Scenario C (N=204) | Scenario D (N=169) |
| --- | --- | --- | --- | --- |
| Predictor variables | Anti-DENV and anti-JEV pre-infection PRNT titres.  Anti-DENV and anti-JEV post-infection PRNT titres.  Anti-DENV and anti-JEV change-in-infection PRNT titres.  The number of days between measurement of the pre- and post-infection titres and the date RT-PCR was conducted. | Anti-DENV and anti-JEV pre-infection PRNT titres.  Anti-DENV and anti-JEV post-infection PRNT titres.  Anti-DENV and anti-JEV change-in-infection PRNT titres.  The number of days between measurement of the pre- and post-infection titres and the date RT-PCR was conducted.  Age and school of participant.  Year of infection. | Anti-DENV post-infection PRNT titres. | Anti-DENV and anti-JEV pre-infection PRNT titres.  Anti-DENV and anti-JEV post-infection PRNT titres.  Anti-DENV and anti-JEV change-in-infection PRNT titres.  The number of days between measurement of the pre- and post-infection titres and the date RT-PCR was conducted. |
| Cases | All cases. | All cases. | All cases. | Cases in seropositive individuals. |
