# Supplementary material for: Predicting the infecting dengue serotype from antibody titre data using machine learning
Source: PLoS Comput Biol. 2024 Dec 23;20(12):e1012188. doi: 10.1371/journal.pcbi.1012188 (PMC11706371; doi:10.1371/journal.pcbi.1012188)
Supplement: S4 Table — For each performance metric, the mean and 95% confidence interval were calculated using 100 bootstrap samples of the hold-out-sample (10%). There were no DENV4 cases in the test set. ANN: artificial neural network. MLR: multinomial logistic regression. SVM: support vector machine. GBM: gradient boosting machine. RF: random forest. DENV: dengue virus. (DOCX) [file pcbi.1012188.s009.docx]

**S4 Table: Mean (95% confidence interval) test class performance of regression and machine learning models for predicting the infecting DENV serotype using all titre predictor variables, plus age, school, and year of infection (Scenario B).** For each performance metric, the mean and 95% confidence interval were calculated using 100 bootstrap samples of the hold-out-sample (10%). There were no DENV4 cases in the test set. ANN: artificial neural network. MLR: multinomial logistic regression. SVM: support vector machine. GBM: gradient boosting machine. RF: random forest. DENV: dengue virus.

| Serotype | Classifier | Prevalence | Sensitivity | Specificity | PPV | NPV |
| --- | --- | --- | --- | --- | --- | --- |
| DENV1 | RF | 15.79% | 0.41 (0.00-1.00) | 0.96 (0.88-1.00) | 0.69 (0.00-1.00) | 0.90 (0.83-1.00) |
|  | GBM |  | 0.58 (0.16-1.00) | 0.95 (0.88-1.00) | 0.73 (0.29-1.00) | 0.93 (0.85-1.00) |
|  | SVM |  | 0.70 (0.33-1.00) | 0.92 (0.75-1.00) | 0.68 (0.27-1.00) | 0.94 (0.87-1.00) |
|  | ANN |  | 0.56 (0.00-1.00) | 0.94 (0.75-1.00) | 0.70 (0.00-1.00) | 0.92 (0.83-1.00) |
|  | MLR |  | 0.62 (0.00-1.00) | 0.93 (0.81-1.00) | 0.67 (0.00-1.00) | 0.93 (0.83-1.00) |
| DENV2 | RF | 52.63% | 0.94 (0.80-1.00) | 0.70 (0.39-0.95) | 0.78 (0.63-0.96) | 0.92 (0.75-1.00) |
|  | GBM |  | 0.93 (0.80-1.00) | 0.80 (0.56-1.00) | 0.84 (0.69-1.00) | 0.92 (0.75-1.00) |
|  | SVM |  | 0.85 (0.60-1.00) | 0.81 (0.56-1.00) | 0.84 (0.68-1.00) | 0.84 (0.67-1.00) |
|  | ANN |  | 0.88 (0.65-1.00) | 0.77 (0.50-1.00) | 0.82 (0.64-1.00) | 0.86 (0.67-1.00) |
|  | MLR |  | 0.81 (0.55-1.00) | 0.79 (0.44-1.00) | 0.82 (0.62-1.00) | 0.81 (0.58-1.00) |
| DENV3 | RF | 31.58% | 0.69 (0.33-1.00) | 0.94 (0.85-1.00) | 0.86 (0.60-1.00) | 0.87 (0.76-1.00) |
|  | GBM |  | 0.71 (0.33-1.00) | 0.92 (0.77-1.00) | 0.82 (0.50-1.00) | 0.88 (0.74-1.00) |
|  | SVM |  | 0.70 (0.33-1.00) | 0.91 (0.77-1.00) | 0.79 (0.56-1.00) | 0.87 (0.75-1.00) |
|  | ANN |  | 0.68 (0.25-1.00) | 0.89 (0.77-1.00) | 0.74 (0.50-1.00) | 0.86 (0.72-1.00) |
|  | MLR |  | 0.62 (0.17-1.00) | 0.87 (0.65-1.00) | 0.71 (0.35-1.00) | 0.84 (0.70-1.00) |
