# Supplementary material for: Predicting the infecting dengue serotype from antibody titre data using machine learning
Source: PLoS Comput Biol. 2024 Dec 23;20(12):e1012188. doi: 10.1371/journal.pcbi.1012188 (PMC11706371; doi:10.1371/journal.pcbi.1012188)
Supplement: S5 Table — (DOCX) [file pcbi.1012188.s010.docx]

**S5 Table: Description of the hyperparameters used to tune each machine learning classifier.**

| Hyperparameters | Range of values tuned over |
| --- | --- |
| RF | |
| *Mtry*: the size of the subset of predictors to split on at each node. This increasing stochasticity and reduces model variance. | 1: The number of predictor variables |
| *Splitting rule*: decides the point at which to the split the variable at each node. For gini, the gini index is used to evaluate the optimal cut point which maximizes the node purity. Gini index: $\sum_{\boldsymbol{k=1}}^{\boldsymbol{K}} \boldsymbol{p}_{\boldsymbol{mk}}\boldsymbol{(}\boldsymbol{1- p}_{\boldsymbol{mk}}\boldsymbol{)}$, where $\boldsymbol{p}_{\boldsymbol{mk}}$ is the proportion of class k observations in node *m*. If extratrees is specified, a random cut point is chosen, rather than local optimal cut-point, increasing model stochasticity. | Gini or extratrees |
| *Number of trees to grow*: RF is robust to overfitting even as the number of trees approaches infinity. | 500 |
| GBM | |
| *Interaction depth*: the maximum number of splits in each tree. A single split fits an additive model, two splits fit a two-way interaction model and so on. In this way, interaction depth controls model complexity. | 1, 3, 5, 7 |
| *Shrinkage*: the learning rate of the boosting algorithm. Shrinkage scales the contribution of each tree by a factor between 0-1. Smaller values tend to increase performance but require more trees to be grown. | 0.001, 0.01, 0.1 |
| *Number of trees to grow*: unlike RF, GBM can result in overfitting as the number of trees increases, although this happens slowly. | 200, 400, 600, 800, 1000 |
| *Minimum number of observations in terminal node*: controls tree complexity. | 5 |
| *Bag fraction*: subsample of the training data available at each iteration to grow the next tree. Increases model stochasticity and reduces variance. | 0.5 |
| SVM | |
| *Degree*: the degree of the polynomial kernel function. Larger values are more likely to overfit the data. | 2, 3, 4 |
| *Cost*: the penalty for violations to the margin of the separating hyperplanes. If cost = 0 and classes are separable, then no misclassifications would be accepted. Theoretically, with enough dimensions any classification problem can be solved, so regularisation via increasing the cost helps to control overfitting by allowing more violations to the margin. | 2 ^-2: 9^ |
| Hyperparameters | **Range of values tuned over** |
| ANN | |
| *Decay*: Adds a penalty to large regression coefficients (pulls the weights towards 0), so any large value must have a significant effect on the model errors to be tolerated. Helps control the regularization process. | 0, 0.1, 0.25, 0.5 |
| *Size*: The number of hidden nodes in the intermediatory layer. Lower values help to control overfitting. | 3:15 |
| *Repeats*: Number of independent models to average over. Helps to reduce variance. | 5 |
